# Spinal muscular atrophy is also a disorder of spermatogenesis

**DOI:** 10.1186/s13023-024-03494-2

**Published:** 2024-12-20

**Authors:** Armelle Magot, Arnaud Reignier, Olivier Binois, Anne Laure Bedat-Millet, Jean-Baptiste Davion, Louise Debergé, Karima Ghorab, Lucie Guyant, Émilie Laheranne, Pascal Laforet, Claire Lefeuvre, Martial Mallaret, Maud Michaud, Chahla Omar, Aleksandra Nadaj-Pakleza, Guillaume Nicolas, Jean Baptiste Noury, Antoine Pegat, Morgane Péré, Emmanuelle Salort-Campana, Guilhem Sole, Marco Spinazzi, Céline Tard, Carole Vuillerot, Yann Péréon

**Affiliations:** 1 Centre de Référence Des Maladies Neuromusculaires AOC, CHU de Nantes, Filnemus, Euro-NMD, Hôtel Dieu, Nantes, France; 2 Service de Médecine Et Biologie de La Reproduction, Gynécologie Médicale, CHU de Nantes, Nantes, France; 3 Service de Biologie de La Reproduction–CECOS, Hôpital Antoine Béclère, AP-HP, Université Paris Saclay, Clamart, France; 4 Centre de Référence Des Maladies Neuromusculaires Nord/Est/Ile de France, Services de Neurologie Et Neurophysiologie, CHU Charles Nicolle, Rouen, France; 5 Centre de Référence Des Maladies Neuromusculaires Nord/Est/Ile de France, CHU Lille, Lille, France; 6 Centre de Référence Des Maladies Neuromusculaires AOC, Service de Neurologie Et Des Maladies Neuromusculaires, CHU de Bordeaux, FILNEMUS, Euro-NMD, Bordeaux, France; 7 Centre de Référence Des Maladies Neuromusculaires AOC, CHU de Limoges, Limoges, France; 8 Service de Neurophysiologie Et Service de Génétique Clinique, CHU de Rouen, Rouen, France; 9 Service de Neurologie, CHU Raymond Poincaré, APHP, Garches, France; 10 Université de Versailles Saint Quentin en Yvelines, Garches, France; 11 Centre de Référence Des Maladies Neuromusculaires, Service de Neurologie, CHU Grenoble Alpes, Université Grenoble Alpes, Inserm, U1216, Grenoble Institut Neurosciences, Grenoble, France; 12 Service de Neurologie, Centre de Référence Maladies Neuromusculaires Nord-Est-Ile de France, CHRU Central, Nancy, France; 13 Centre de Référence Des Maladies Neuromusculaires Nord/Est/Ile de France, Service de Neurologie, Hôpitaux Universitaires de Strasbourg, EURO-NMD, Paris, France; 14 Centre de Référence Des Maladies Neuromusculaires AOC, Inserm, LBAI, UMR1227, CHRU de Brest, Brest, France; 15 Service ENMG Et de Pathologies Neuromusculaires, Centre de Référence Des Maladies Neuromusculaires PACA-Réunion-Rhône Alpes, Hôpital Neurologique P. Wertheimer, Hospices Civils de Lyon, Lyon, France; 16 Plateforme de Méthodologie Et de Biostatistique, Centre Hospitalier Universitaire de Nantes, Nantes, France; 17 Centre de Référence PACA Réunion Rhône Alpes, AP-HM, Hôpital La Timone, FILNEMUS, Marseille, France; 18 Centre de Référence Des Maladies Neuromusculaires AOC, Service de Neurologie, CHU d’Angers, Angers, France; 19 Centre de Référence Des Maladies Neuromusculaires Nord/Est/Ile-de-France, Service de Neurologie, U1172, CHU de Lille, Lille, France; 20 Centre de Référence PACA Réunion Rhône Alpes, Hospices Civils de Lyon, Hôpital Femme-Mère-Enfant, L’Escale, Service de Médecine Physique Et de Réadaptation Pédiatrique, Bron, France; 21 NeuroMyogen Institute, CNRS UMR 5310–INSERM U1217, University of Lyon, Lyon, France

## Abstract

**Background:**

Spinal muscular atrophy (SMA) patients benefit from pre-mRNA splicing modifiers targeting the *SMN2* gene, which aims to increase functional SMN production. The animal toxicity affecting spermatogenesis associated with one such treatment raised questions about male SMA patients’ spermatogenesis.

**Methods:**

This descriptive, cross-sectional study was conducted from June 2022 to July 2023. The study involved adult male patients with genetically confirmed SMA type 2 (SMA2) or SMA3 from 13 French neuromuscular centers. The patients’ general data, motor severity, urological history, exposure to certain factors, parenthood, and spermogram results were obtained. All patients were enrolled prior to exposure to risdiplam.

**Findings:**

Sixty-eight patients were enrolled ( 36 SMA2 and 32 SMA3 patients). Forty-one patients had fertility data (parenthood history and spermogram analyses) and underwent 33 spermograms. Fertility disorders were identified in 27 of the 41 patients (65·9%, 95%CI 51·3–80·4%) in particular SMA2 patients: 19 cases (90^.^5%, CI 77·9–100%) (SMA3: 8 cases (40%, CI 18·5–61·5%). Among the patients with available spermograms, 81% (27/33) had abnormal sperm concentration; 30% presented azoospermia. These abnormalities were significantly associated with SMA type (more prevalent in SMA2 patients, *p* < 0·001), disease motor severity, which included age at the loss of walking ability and wheelchair use duration (*p* < 0·001). The Motor Function Measure (MFM) determined that the sperm counts were also correlated with disease severity (*p* < 0·01).

**Interpretation:**

The fertility disorders were correlated with SMA severity and were particularly evident in SMA2 patients. In the latter, sperm concentration positively correlated with MFM. This study is the first one to link fertility disorders with spermogram abnormalities in SMA males. Understanding spermatogenesis in SMA is crucial, especially with new therapies such as risdiplam. Consequently, conducting systematic spermogram studies prior to SMA treatment is recommended.

## Introduction

Spinal muscular atrophy (SMA) is a rare autosomal recessive neuromuscular disorder characterized by the progressive loss of lower motor neurons, which leads to diffuse muscle weakness. SMA occurs due to deletion or mutations in the survival of motor neuron 1 gene (*SMN1*) [1]. Most humans possess a second closely related gene, *SMN2*. However, exon 7 skipping in *SMN2* RNA primarily generates an unstable SMNΔ7 protein that undergoes rapid degradation. Consequently, the quantity of full-length SMN protein produced by *SMN2* is inadequate to offset the *SMN1* deficiency. The number of *SMN2* copies varies across individuals. SMA patients are typically classified into five types (type 0–4) according to the best-acquired motor function and age of onset. The *SMN2* copy number determines the phenotype severity and is partially correlated to the SMA type [1]. In France, individuals categorized as SMA type 0 (SMA0) and SMA1 had extremely low survival rates prior to the introduction of disease-modifying therapies.

Currently, in addition to *SMN1* gene therapy, the specific SMA treatments include nusinersen or risdiplam [2, 3]. Risdiplam is an *SMN2* pre-mRNA splicing modifier that promotes exon 7 inclusion and full-length *SMN2* mRNA production via its ability to bind two sites within exon 7 of the *SMN2* transcript (exonic splicing enhancer 2 and 5′ splice site) [2, 4, 5]. Comprehensive toxicity investigations focusing on the effects of *SMN2* splicing modifiers on the reproductive system revealed that the risdiplam resulted in qualitative and quantitative anomalies in spermatogenesis in both rats and monkeys: In monkeys treated with RG7800 (closely related *SMN2* mRNA-splicing compound), no damage to spermatogonia was observed, and the testicular changes were fully reversible after an eight-week recovery. In rats, seminiferous tubule degeneration occurred, with full reversibility in about half of the rats following a recovery period after exposure to risdiplam or RG7800 [6]. The effect of risdiplam on human spermatogenesis has not been assessed (as the phase 3 study was designed for children). It is anticipated that the effects on the male reproductive system would also be reversible in humans after a complete spermatogenic cycle, which typically lasts ~ 70 days. Consequently, the restoration of regular fertility function would occur within 70 days, in addition to six half-lives of the drug, after the final dose. Given this rationale, guidelines have been established in both the US Prescribing Information and the European Summary of Product Characteristics for male SMA patients considering the possibility of conception [6, 7].In France, multidisciplinary expert discussions led to the proposal for systematic cryopreservation before risdiplam treatment introduction.

The estimated male infertility rate in the general European population is 7·5% [8, 9], with 30% of cases considered idiopathic infertility. Among the recognized etiologies, testicular dysfunction is the most frequent cause of disturbed spermatogenesis (cryptorchidism, varicoceles, cytotoxic drugs). Genetic factors, such as chromosomal abnormalities and gene deletions, significantly influence male infertility [10]. Recent advances in genome-wide techniques and next-generation sequencing (NGS) have helped identify infertility-related genes. [11]

The potential adverse effect of risdiplam on spermatogenesis raised questions about fertility in SMA, which has been little studied [12]. Considering the potential coexistence of acquired male infertility factors in SMA, differentiating them from being a component of SMA disease manifestations would be crucial. Accordingly, this study aimed to determine whether male SMA patients have fertility disorders, and if so, to identify the factors associated with these disorders.

## Methods

### Study design

This cross-sectional descriptive study was conducted from June 2022 to July 2023 in France. The data were collected during the patient’s routine visit to their neuromuscular reference center. The relevant French non-interventional research committee (GNEDS) approved the study.

### Patients

Inclusion in the study was proposed to all adult male patients with a genetically confirmed diagnosis of SMA who were followed at the French Health Care Network for rare neuromuscular diseases (Filnemus). Only patients with SMA2 and SMA3 were included. SMA1 patients were not included as exceptionally few SMA1 patients have reached adulthood to date. SMA4 patients were also excluded because none of the treatments currently have marketing authorization for this indication.

The collected data were: age, SMA type, *SMN1* genetic abnormality, *SMN2* copy number, height, weight, recent motor function data [Motor Function Measure, MFM [13] and Revised Upper Limb Module, RULM [14]], ambulant status, wheelchair use duration, history of diabetes mellitus, and substance use (tobacco, alcohol, cannabis). Regarding urology, information on cryptorchidism, hypospadias, testicular torsion, orchitis, or other urological histories was requested. We queried the age of puberty, or if not available, the age of the first shave. The patients were queried about parenthood, and if applicable, age at the birth of the first child and whether they had experienced procreation difficulties (> 12 months without conceiving). Previous, ongoing, or planned exposures to SMA treatments (risdiplam, nusinersen, salbutamol) were documented. Furthermore, data from the sperm analysis of patients who underwent a spermogram before starting risdiplam treatment (volume, pH, sperm count, round cell count, motile sperm percentage) were collected. Moreover, the semen collection method was also queried. Some sperm analyses conducted as part of infertility assessments were retrospectively included.

### Statistical analysis

The data were analyzed using the online tool biostatTGV (https://biostatgv.sentiweb.fr/). Quantitative data are presented as the mean and standard deviation (SD) and compared between groups with the Mann–Whitney test. Categorical data are presented as the exact number and percentage and compared between groups with the χ^2^ test or Fisher’s exact test if necessary. Correlations between various parameters were determined using Spearman correlations. A *p*-value ≤ 0.05 was considered significant. The statistical power for the primary outcome was 0.976.

## Results

### ***Total cohort (***Table 1***)***

**Table 1:** Main features of the population. MAR: Medically assisted reproduction

	SMA2 (n = 36)	SMA3 (n = 32)	*p*
Age at inclusion (years), mean ± SD	32·34 ± 7·6	41·2 ± 12·0	< 0·001
*SMN2* * copies, n (%)*			
1	1 (2·7%)	0 (0%)	0·32
2	1 (2·7%)	0 (0%)	0·32
3	21 (58·3%)	6 (18·8%)	< 0·0001
4	1 (2·7%)	17 (53·1%)	< 0·0001
Height (cm), mean ± SD	158·2 ± 9·5 (n = 30)	170·0 ± 9·8 (n = 27)	< 0·0001
Weight (kg), mean ± SD	56·4 ± 19·3 (n = 33)	77·2 ± 23·2 (n = 31)	< 0·0005
Diabetes mellitus, n (%)	0 (0%)	2 (6·3%)	0·49
Last MFM (%), mean ± SD	16·5 ± 11·9 (n = 23)	49·5 ± 19·4 (n = 28)	< 0·0001
Last RULM (%), mean ± SD	7·8 ± 7 (n = 20)	21·2 ± 11·8 (n = 21)	< 0·0001
Urologic history, n (%)	7 (19·4%)	3 (9·4%)	0·24
Cryptorchidism	3 (8·3%)	2 (6·3%)	> 0·99
With surgery	2 (5·6%)	2 (6·3%)	
Hypospadias	0 (0%)	1 (3·1%)	> 0·99
Orchitis	1 (2·8%)	0 (0%)	> 0·99
Varicocele	1 (2·8%)	0 (0%)	> 0·99
Testicular torsion	2 (5·6%)	0 (0%)	0·90
Substance consumption, n (%)	8 (22·2%)	1 (3·1%)	0·020
Alcohol	3 (8·3%)	0 (0%)	0·09
Tobacco	6 (16·7%)	1 (3·1%)	0·07
Cannabis	3 (8·3%)	1 (3·1%)	0·36
Age of puberty, mean ± SD	14·0 ± 2·1 (n = 15)	13·5 ± 0·9 (n = 18)	0·70
History of fatherhood, n (%)	8 (22·2%)	13 (40·6%)	0·10
Spontaneous pregnancy	1 (2·8%)	10 (31·3%)	< 0·005
Age at birth of first child (years), mean ± SD	33·7 ± 5·5	31·8 ± 5·9	0·64
Known hypofertility or sterility, n (%)	11 (30·6%)	5 (15·6%)	0·15
No offspring	5 (45·4%)	2 (40%)	0·84
Successful MAR	6 (54·5%)	2 (40%)	0·59
Adoption	0 (0%)	1 (20%)	0·14
*Ongoing treatment, n (%)*			
None	18 (50%)	11 (34·4%)	0·19
Nusinersen	5 (13·9%)	11 (34·4%)	< 0·05
Risdiplam	12 (33·3%)	9 (28·1%)	0·64
Salbutamol	1 (2·8%)	3 (9·4%)	0·25

This study included 68 patients from 13 French neuromuscular centers. The mean age at inclusion was 36.6 years (range, 20–65 years). Thirty-six patients (53%) had SMA2, and 32 patients (47%) had SMA3. All patients had a homozygous deletion of *SMN1*. Table 1 presents the main features of both groups. As expected, both groups demonstrated significantly different *SMN2* copy numbers, weight, height, and motor scales. Spontaneous parenthood (pregnancy within less than 12 months without medically assisted reproduction) was significantly more common among the SMA3 patients. The SMA2 patients more frequently had a history of known infertility than the SMA3 patients (30% *vs*. 15%), although this did not reach statistical significance.

Twenty-three SMA2 patients were approached for sperm preservation before risdiplam introduction. Eight of the 23 patients declined (35%) for the following reasons: four did not wish to have children, one had a history of known azoospermia, two had cryopreserved sperm samples or a gestational surrogacy plan, and one provided no explanation. Fourteen SMA3 patients were approached for sperm preservation before risdiplam introduction. Three of the 14 patients declined (21%): two did not wish to have children and one eventually did not follow through with the treatment proposal. There was no statistically significant difference between the patients who underwent spermograms and those who did not.

Similarly, there was no significant difference between the patients who underwent a spermogram and the rest of the cohort.

### ***Spermograms cohort (***Table 2***)***

**Table 2:** Semen analysis data according to the World Health Organization (WHO) laboratory manual for the examination and processing of human semen, 5th edition[37]

	SMA2 (n = 20)	SMA3 (n = 13)	p
Collection method, n (%)			
Self-collection	2 (10%)	8 (61.5%)	< 0·0001
Assistance from a third person	6 (30%)	0 (0%)	< 0·05
Penile vibratory stimulation	5 (25%)	0 (0%)	0·06
Testicular sperm extraction	2 (10%)	0 (0%)	0·28
Unknown	5 (25%)	5 (38.5%)	0·41
Effective cryopreservation, n (%)	7 (35%)	8 (61·5%)	0·13
Semen analysis			
Normal volume (> 1.5 ml)	12 (86%; n = 14)	11 (100%; n = 11)	0·19
Abnormal spermogram, n (%)	19 (95%)	8 (61%)	< 0·05
Azoospermia	9 (45%)	1 (7.7%)	< 0·05
Oligozoospermia < 2.5 M/ml	8 (26.7%)	4 (30·7%)	0·72
Progressively motile at 1 h (%), mean ± SD	16·3 ± 17·4 (n = 4)	40 ± 0 (n = 1)	
Oligozoospermia < 15 M/ml	1 (1%)	3 (23%)	0·30
Progressively motile at 1 h (%), mean ± SD	37 ± 0 (n = 1)	20.0 ± 10·0 (n = 3)	
Necroteratozoospermia	1 (0·5%)	0 (0%)	0·41
Normal > 15 M/ml	1 (0·5%)	5 (38·4%)	< 0·05
Progressively motile at 1 h (%), mean ± SD	18 ± 0 (n = 1)	47·2 ± 17·7 (n = 5)	

During the study, a total of 26 patients underwent sperm analysis before risdiplam introduction. Furthermore, the semen analysis results of seven patients who had undergone the analysis earlier due to infertility concerns were collected retrospectively. All the patients underwent semen analysis before starting any disease modifier treatment except 2 of them treated by salbutamol and one by nusinersen. Hence, the semen analysis data of 33 patients (20 SMA2 patients and 13 SMA3 patients) were collected. Table 2 presents the sperm analysis results and collection methods.

The semen collection method analysis highlighted a significant difference for the SMA3 patients, who were more likely to have self-collected samples due to less motor impairment. The sperm concentration was abnormal in 95% of the SMA2 patients, while 61% of SMA3 patients had abnormal sperm concentration [estimated at 7.5% in European populations [8, 9]. Azoospermia was significantly more frequent in the SMA2 patients rather than in SMA3 patients (45% vs. 7.7%). Conversely, the SMA3 patients tended to exhibit normal sperm parameters more frequently than the SMA2 patients (38.4% vs. 1%).

### ***Subgroups Comparison (***Figs. 1 and 2***, ***Table 3***)***

**Fig. 1:**
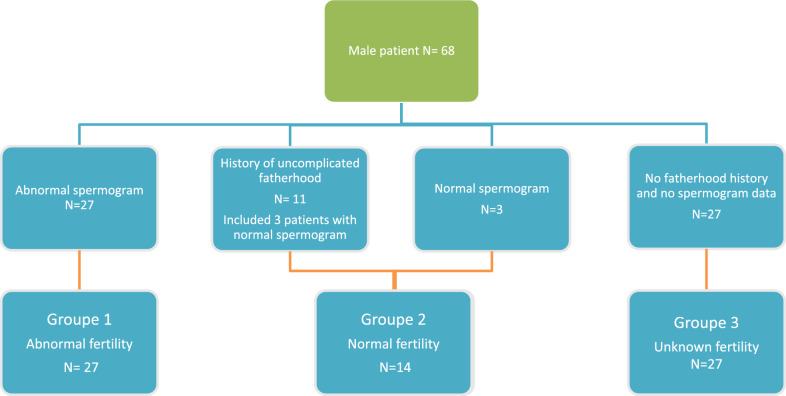
Flow chart of patient division into the three groups

**Fig. 2:**
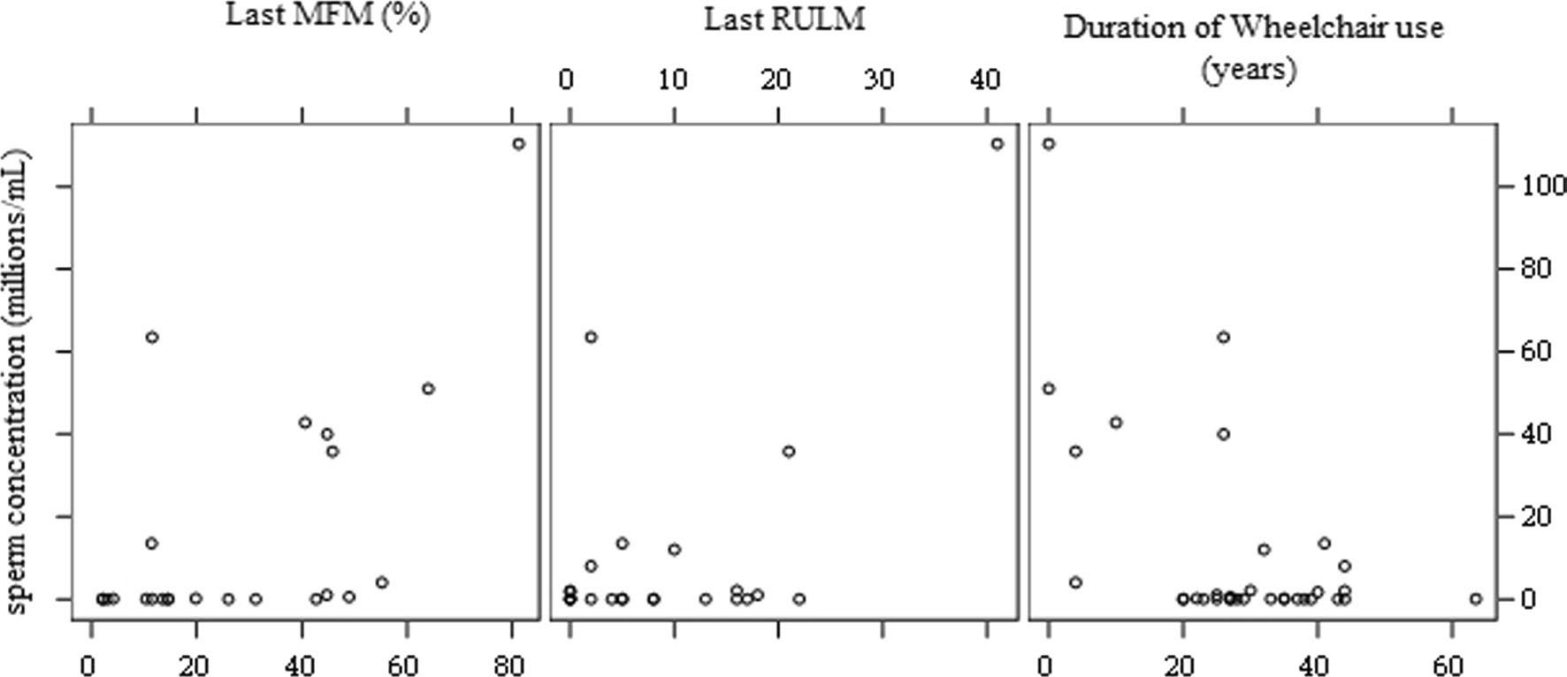
Scatter Plot of correlations between sperm concentration with MFM, RULM and duration of wheelchair use

**Table 3:** Comparison between groups 1 (fertility disorders) and 2 (normal fertility)

	Fertility disorder (n = 27)	Normal fertility (n = 14)	p
Age at inclusion (years), mean ± SD	36·0 ± 10·6	42·9 ± 12·4	0·06
SMA2, n (%)	19 (70·4%)	2 (14·3%)	0·0007
SMA3, n (%)	8 (29·6%)	12 (85·7%)	0·0007
SMN2 copy number, mean ± SD	3·05 ± 0·6 (n = 19)	3·6 ± 0·7 (n = 10)	0·0200
Height (cm), mean ± SD	159·4 ± 8·3 (n = 24)	176·6 ± 6·6 (n = 10)	< 0·0001
Weight (kg), mean ± SD	62·3 ± 22·7 (n = 27)	88·5 ± 23·6 (n = 13)	0·010
Age at wheelchair use (years), mean ± SD	3·5 ± 5·8 (n = 27)	24·4 ± 14·0 (n = 9)	0·0005
Duration of wheelchair use (years), mean ± SD	32·4 ± 11·2	11·7 ± 12·9	0·0003
Last MFM (%), mean ± SD	20·9 ± 17·5 (n = 17)	48·2 ± 18·4 (n = 14)	< 0·0005
Last RULM, mean ± SD	7·6 ± 7·1 (n = 20)	23·1 ± 12·9 (n = 8)	< 0·005
Diabetes mellitus, n (%)	1 (3·7%; n = 26)	1 (7·1%; n = 13)	> 0·99
Urologic history, n (%)			
Cryptorchidism	2 (7·4%; n = 6)	1 (7·1%; n = 13)	> 0·99
Substance consumption, n (%)			
Tobacco	5 (18·5%)	0 (0%)	0·14
Cannabis	2 (7·4%)	0 (0%)	> 0·99
Age of puberty, mean ± SD	13·9 ± 1·7 (n = 15)	13·4 ± 1·1 (n = 5)	0·62

The patients were subsequently divided into three groups (Fig. 1): Group 1, SMA patients with sperm analysis abnormalities in terms of spermatozoid count (< 15 M/ml) (n = 27, including 15 patients with known fertility issues in their parenthood plan); Group 2, patients who had children without conception problems (n = 8) or had a normal sperm analysis during the assessment (n = 6); Group 3, patients without a sperm analysis and no history of children, rendering their fertility inconclusive (n = 27). Excluding Group 3, fertility disorders were present in 65.9% (*95%CI 51.3–80.4%*) of the patients, in particular in SMA2 with 19 cases (9.^.^5%, *95CI 77.9–100%*) while 8 patients with SMA 3 were concerned (40%, *95CI 18.5–61.5%*).

Subsequently, Group 1 was compared with Group 2 to determine the presence of factors associated with fertility disorders (Table 3).

The presence of fertility disorders was linked to SMA severity: the risk of experiencing fertility issues was highest in patients with SMA2, low *SMN2* copy numbers, low weight, short stature, and lower MFM and RULM scores. Fertility disorders were not correlated with the presence of diabetes mellitus, height, history of cryptorchidism, toxic substance use, or age of puberty. The spermatozoid concentration was significantly positively correlated with severity as measured by the MFM (Spearman correlation coefficient: 50.8%, *95%CI 18.7–73.1%*; *p* = 0.01) (Fig. 2). The RULM and wheelchair use duration were not significantly correlated.

## Discussion

To our knowledge, this is the first study to demonstrate fertility disorders in relation to spermogram abnormalities in male SMA patients. Fertility disorders affect ~ 40% of our SMA population, a significantly higher rate than compared to the prevalence in the general population (estimated at 7.5% in European populations [8, 9]). In the present cohort, the SMA2 patients had a higher frequency of fertility disorders, which was also correlated with lower *SMN2* copy numbers, lower weight, shorter height, and lower MFM or RULM scores. Unfortunately, the sample size did not allow a multivariable logistic regression to be conducted, which could have enabled the differentiation of non-independent variables. Nevertheless, factors such as weight, height, *SMN2* copy number, and the MFM and RULM scores were logically linked to the SMA type and were not independent variables that accounted for fertility. Indeed, MFM revealed a positive correlation between sperm count and SMA severity, where an increased MFM score was accompanied by increased sperm concentration. Although fertility disorders are typically present in all SMA types, our results demonstrated their association with disease severity.

Few SMA studies have focused on fertility issues, and little is known about the non-muscular phenotypes in SMA patients. A US database study of the insurance claims of 1038 SMA patients broadened the phenotype spectrum as compared to controls, particularly in the patients with longer lifespans and who reached reproductive age (SMA4 is diagnosed between the ages of 21 and 65 years)[12]. Significantly, observable reproductive system phenotypes, including testicular hypofunction, affected 9.5% of the patients, and infertility was noted in 2.6% of male SMA cases. Within this group, fertility issues might notably serve as the primary reason for seeking medical consultation before the onset of neuromuscular symptoms.

Cryptorchidism is a reliable predictive indicator of male infertility in several infertility cases [15]. An SMA cohort demonstrated more patients with cryptorchidism (1/3 of 26 male patients), particularly for SMA1 (60%) and SMA2 (30%) [16]. Indeed, some older observations reported atrophic testes or hypofertility, but genetic confirmation was not used at the time [17]. Furthermore, the idea that the patients presented X-linked spinal and bulbar muscular atrophy (Kennedy disease) could not be excluded [18]. The present study did not establish a link between the presence of cryptorchidism and fertility disorders.

Our results clearly demonstrated a link between disease severity and fertility disorders. SMA2 patients were more frequently affected by spermatogenesis disorders, and the sperm count correlated with the motor scale scores. This link to the degree of disability raises questions about the effect of the sitting position. Inadequate thermoregulation of scrotal temperature causes testicular hyperthermia, which negatively impacts spermatogenesis and lowers sperm quality. Heat stress triggers germ cell apoptosis, particularly affecting spermatocytes and spermatids, though the exact mechanism is unclear. The scrotal sac helps regulate temperature through its structure, and prolonged immobility, such as sitting or reclining for extended periods, raises testicular temperature [19]. Following at least 20 min of sitting, men with spinal cord injury (SCI) exhibited higher deep scrotal temperatures and less mobile sperm compared to non-disabled men [20]. On the contrary, cooling interventions did not demonstrate normalized semen parameters, and ambulatory men with SCI exhibited similar semen abnormalities [21]. In these patients, abnormal semen analysis parameters were characterized by normal sperm concentration but significantly lower sperm motility and viability [22]. Other mechanisms to explain these sperm motility anomalies are discussed but without formal proof or demonstration, *e.g.* anejaculation, role of seminal plasma [23–25]. To our knowledge, there is no published research on the fertility of patients requiring wheelchairs during their youth [*e.g.*, Duchenne muscular dystrophy (DMD) patients]. A recently published study of a DMD population highlighted the benefits of early testosterone treatment, which improved the boys’ testicular volume. Nevertheless, the DMD testicular volume remained lower than that of normal boys of the same age. However, there was no concurrent analysis of spermograms, and the major distinctiveness of these patients is that they were treated early and chronically with corticosteroids, which added confounding factors [26].

Another explanation for the fertility disorders in our population is the proper role of SMN. SMN protein has several roles and is not restricted to motor function [27]. The absence of SMN leads to the swift differentiation of *Drosophila* germline stem cells and mouse embryonic stem cells underscoring the significance of SMN in germ cell development and stem cell biology [28, 29]. SMA mouse models demonstrated stunted testicular tissue growth, accompanied by gamete development abnormalities and the loss of the spermatogonia-specific marker [30]. Mild SMA mice have extensive seminiferous tubule degeneration, loss of post-meiotic cells, and substantially decreased male fertility. Indeed, the testis exhibits the most pronounced SMN expression compared to all other investigated tissues, implying a sustained need to facilitate diverse aspects of testis development and function [31].

In contrast to its behavior in other tissues, *SMN2* exon 7 is predominantly integrated within the testis [31]. This suggests that the transition in *SMN2* exon 7 splicing fulfills, at least partially, the demand for heightened SMN expression in the testes. One hypothesis states that *SMN2* retention throughout evolution was driven by the heightened demand for SMN in male reproductive organ development [31]. Supplementary mechanisms, such as increased transcription and translation, could also be involved in the elevated SMN expression observed in mammalian testes. Studies of SMA mouse model muscles and neurons revealed the disruption of various signaling cascades implicated in the proliferation of Sertoli cells, which nourish germ cells [32]. Finally, enhancing SMN expression improved spermatogonia stem cell propagation and facilitated spermatogenesis [33].

Cumulative evidence supports the effect of sex on SMN functions as well as on the pathogenesis of SMA [32]. In animals, recent studies have demonstrated a gender-specific improvement in SMA pathology following a modest peripheral increase in SMN levels, particularly highlighting the effects on male reproductive health. Early administrations of SMA treatments significantly enhanced testicular development and spermatogenesis in male mice [34, 35]. The findings suggest that low SMN levels during early stages of testicular development lead to long-term disruptions in gene expression, exacerbating male reproductive issues. Moreover, T-cell-restricted intracellular antigen 1 (TIA1) has been identified as a crucial regulator of SMN exon 7 splicing, further implicating gender-specific modifiers in SMA pathology [36]. These results emphasize the need to consider sex in developing treatment strategies, with a particular focus on reproductive organs in clinical settings, as gender-specific effects become increasingly evident.

One limitation of our study is the potential recruitment bias, with recruitment non-exhaustiveness within the various involved centers. It is most likely that most of the included patients underwent sperm analysis, primarily patients scheduled to receive risdiplam treatment, consequently frequently representing more severe cases. We were also limited by the inability to conclude on the fertility of a portion of the population due to either the lack of sperm analysis or the absence of a desire for parenthood. Another limitation was that we were unable to explore the hypothesis regarding the effect of sitting position on disease severity.

## Conclusion

The results demonstrated that male SMA patients exhibit high infertility levels, which were correlated with disease severity. The most probable hypothesis is of a specific role linked to the absence of SMN in the genesis of these disorders, which is supported by animal studies. Nevertheless, comparing the spermogram analyses of patients with another neuromuscular disease requiring wheelchairs during their youth to that of SMA patients with the same or similar disease severity could be an interesting means of understanding the role of the sitting position or the SMN protein. The question of the long-term effects of modifying treatments, particularly the effectiveness of risdiplam, will also arise. A systematic study of spermograms in young adult and adult SMA patients before and after treatment would be appropriate.

## Data Availability

The data that support the findings of this study are not openly available due to reasons of sensitivity and are available from the corresponding author upon reasonable request. Data are located in controlled access data storage at Nantes Hospital University: U:\mes documents\Documents\protocoles\FERMASI.
